# High Bak Expression Is Associated with a Favorable Prognosis in Breast Cancer and Sensitizes Breast Cancer Cells to Paclitaxel

**DOI:** 10.1371/journal.pone.0138955

**Published:** 2015-09-25

**Authors:** Yanwei Luo, Xinye Wang, Heran Wang, Yang Xu, Qiuyuan Wen, Songqing Fan, Ran Zhao, Shihe Jiang, Jing Yang, Yukun Liu, Xiayu Li, Wei Xiong, Jian Ma, Shuping Peng, Zhaoyang Zeng, Xiaoling Li, Joshua B. Phillips, Guiyuan Li, Ming Tan, Ming Zhou

**Affiliations:** 1 The Affiliated Tumor Hospital of Xiangya Medical School, Cancer Research Institute, Central South University, Changsha, Hunan, 410013, P. R. China; 2 Mitchell Cancer Institute, University of South Alabama, Mobile, Alabama, 36604, United States of America; 3 The Second Xiang-Ya Hospital, Central South University, Changsha, Hunan, 410011, P. R. China; 4 The Third Xiang-Ya Hospital, Central South University, Changsha, Hunan, 410013, P. R. China; University of Navarra, SPAIN

## Abstract

Breast cancer has become the leading cause of cancer-related death among women. A large number of patients become resistant to drug chemotherapy. Paclitaxel (Taxol) is an effective chemotherapeutic agent used to treat cancer patients. Taxol has been widely used in human malignancies including breast cancer because it can stabilize microtubules resulting in cell death by causing an arrest during the G2/M phase of the cell cycle. Pro-apoptotic Bcl-2 antagonist killer 1 (Bak) plays an important role in Taxol-induced apoptosis in breast cancer. In our present study, we investigated the expression of the Bak protein and clinicopathological correlations in a large sample of breast cancer tissues by immunohistochemistry. We found that the percentage of high scores of Bak expression in breast cancer was significantly lower than that of the non-cancerous breast control tissue. In addition, lower Bak expression was positively associated with the clinical TNM stage of breast cancer with a significant decrease in overall survival compared with those with higher Bak expression especially in the Luminal and HER2 subtypes. Importantly, higher Bak expression predicted a favorable clinical outcome in the cases treated with Taxol indicated by a higher overall survival than that of patients with lower Bak expression especially in Luminal and HER2 subtypes. Furthermore, these results were confirmed *in vitro* since overexpression of Bak sensitized breast cancer cells to Taxol by inhibiting proliferation and promoting apoptosis; in contrast, downregulation of Bak through siRNA transfection inhibited Taxol induced-apoptosis. Therefore, our results demonstrate that Bak acts as a sensitive biomarker and favorable prognostic factor for Taxol treatment in breast cancer. The restoration of Bak expression would be therapeutically beneficial for Taxol resistant breast cancer patients.

## Introduction

Drug resistance has become a major problem in cancer treatment [1]. Paclitaxel (Taxol), a member of the taxane class of anti-neoplastic microtubule damaging agents widely used in human malignancies like breast cancer, can stabilize microtubules and subsequently cause cell death by arresting the cell cycle at G2/M [2]. However, breast cancer cells frequently develop resistance to Taxol because of the evasion of apoptosis [3]. Breast cancer has become the leading cause of cancer-related death among women with the incidence increasing year by year. Even if a small proportion of the breast cancer patients are resistant to drug chemotherapy, it substantially affects a large number of patients [4]. Thus, overcoming resistance to chemotherapy in breast cancer is one of the major issues in the management of breast cancer patients. Therefore, it is necessary to identify useful biomarkers that distinguish sensitive patients and those resistant to drug treatment.

Pro-apoptotic Bcl-2 antagonist killer 1 (Bak) is a pro-apoptotic member of Bcl-2 family that contains multiple domains. It was reported that the Bcl-2 family-dependent mitochondrial apoptotic pathway was activated in cancer cells during Taxol treatment [5]. Upregulation of Bak by gene transfer can accelerate growth factor deprivation induced-apoptosis in murine lymphoma [6], lung cancer [7], and breast cancer cells [8]. Moreover, knockout of Bak results in multidrug resistance [9]. Furthermore, Anna V. Miller et al. showed that Bak was the mediator of Paclitaxel-induced apoptosis [10]. Our previous study also demonstrated that miR-125b suppressed the expression of Bak, and subsequently inhibited Taxol-induced apoptosis in MDA-MB-231 cells [11], which highlighted its critical role in apoptosis.

However, an association between the expression of Bak proteins and clinicopathological features, prognostic implications, and therapeutic strategies in a large sample of breast cancer tissues has not been reported. In our present study, we investigated the expression of the Bak protein and clinicopathological correlations in breast cancer tissues by immunohistochemistry. We found that percentage of high scores of Bak expression in breast cancer was significantly lower than in the non-cancerous breast control tissue. In addition, lower expression of Bak was positively associated with the clinical TNM stage of breast cancer with a significant decrease in overall survival compared with those with higher expression of Bak especially in the Luminal and HER2 subtypes. Importantly, higher Bak expression predicted a favorable clinical outcome in cases treated with Taxol especially in Luminal and HER2 subtypes. Furthermore, these results were confirmed *in vitro*. We also found that overexpression of Bak sensitized breast cancer cells to Taxol by inhibiting proliferation and promoting apoptosis; in contrast, downregulation of Bak through siRNA transfection inhibited Taxol induced-apoptosis. Therefore, our results demonstrate that Bak acts as a sensitive biomarker and favorable prognostic factor for Taxol treatment in breast cancer. Restoration of the Bak expression will be therapeutically beneficial for Taxol resistant breast cancer patients.

## Materials and Methods

### Tissue samples and clinical data

Two hundred and twenty-five (225) cases of paraffin-embedded breast cancer and sixty-two (62) paraffin-embedded non-cancerous breast tissue samples from November 2001 to September 2012 were obtained from the Second Xiangya Hospital of Central South University. All the tissue samples were constructed into nine slices of tissues microarray as previously described [12] with each sample in duplicate or triplicate. Clinicopathologic characteristics of breast cancer patients were recorded including name, gender, age, occupation, race, clinical tumor node-metastasis (TNM) stage, recurrence, pathology diagnosis, molecular subtype, chemoradiotherapy strategies, telephone number, and address. All the patient information was anonymized and de-identified prior to analysis. The profile of clinicopathologic characteristics of the breast cancer patients is shown in Table 1. Patients’ age ranged from 23 to 71 years old. All of the 225 breast cancer patients had valid follow-up data, and the longest survival time was 121 months. The overall survival (OS) was defined as the time from diagnosis to the date of death or the date last known alive. This study was approved by the Committee on the Ethics of Central South University. All of the individuals participating or their dependents have written informed consent.

**Table 1:** Clinicopathologic characteristics of breast cancer patients.

Age	
Mean±SD	46±0.66
Sex (%)	
Male	0.4%(1/225)
Female	99.6%(224/225)
Tumor size (%)	
T1-T2	56.4%(127/225)
T3-T4	43.6%(98/225)
Nodal metastasis (%)	
Present	76.0%(171/225)
Absent	24.0%(54/225)
Distant metastasis (%)	
Present	10.7%(24/225)
Absent	89.3%(201/225)
TNM stage (%)	
I	9.8%(22/225)
II	54.2%(122/225)
III	25.3%(57/225)
IV	10.7%(24/225)

### Cell culture

The human breast cancer cell lines, MCF-7 and MDA-MB-231, were kindly provided by Dr. Ming Tan who purchased them from ATCC (catalogue numbers: HTB-22™ for MCF7, HTB-26™ for MDA-MB-231) on Sep 17, 2009.These cells were cultured in DMEM(Invitrogen Life Technologies, Carlsbad, CA, USA) supplemented with 10% fetal bovine serum (FBS) (Biological Industries Israel Beit-Haemek Ltd. Kibbutz Beit Haemek, Israel) at 37°C in a humidified incubator containing 5% CO_2_.

### Plasmid construction and transfection

The plasmid pIRES2/GFP/Neo-Bak was constructed in our laboratory. The plasmid pIRES2/GFP/Neo-NC was used as a negative control (NC). MCF-7 cells were transfected with these two plasmids respectively by using Lipofectamine 3000 (Life technologies, Grand Island, NY) in accordance with the manufacture’s instruction. Cells were then incubated at 37°C with 5% CO_2_ for 48 h. The cells were then screened by using G418 (Sigma-Aldrich, St. Louis, MO, USA). The positive clones that highly expressed the Bak gene were selected to culture for further analysis.

In order to knock down the expression of Bak, we transfected small interfering RNA (siRNA) into MDA-MB-231 cells by simultaneously using the following three sequences: siRNA Bak #1: 5’ CCAGUUUGUGGUACGAAGA dTdT3’; siRNA Bak #2, 5‘CAGAGAAUGCCUA UGAGUA dTdT 3‘; siRNA Bak #3, 5‘ CCGACGCUAUGACUCAGAG dTdT 3‘. And siRNA scramble was used as negative control (cat no: Q000000578-1-B, RiboBio Co., Ltd, Guangzhou, China). MDA-MB-231 cells were transfected with the pool of these siRNA sequences by using Lipofectamine 3000 (Life technologies, Grand Island, NY) in accordance with the manufacture’s instruction. After 48 h transfection, the cells were used for further analysis.

### Immunohistochemical staining

Slides were deparaffinized with xylene twice (each for 10 min) and hydrated through an ethanol gradient (100%, 95%, 90%, 80% and 70%, each for 5 min), and rinsed with phosphate-buffered saline (PBS). Bak antigen was retrieved by citric acid buffer (pH6.0) microwave antigen retrieval (high power for 3 min and low power for 15 min).The slides then were treated with 3% H_2_O_2_ for 15 min and washed by tris-buffered saline containing 1% Tween 20 (TBST) for 15 min. Following a 30 min blocking with normal goat serum (Maixin, Fujian, China), slides were incubated with mouse monoclonal anti-Bak (Cell Signaling Technology, Danvers, MA; 1:500 dilution) overnight at 4°C. On the next day, slides were washed three times with TBST (each for 15 min). Slides were then incubated with a secondary anti-mouse antibody (Maixin, Fujian, China) for 90 min at 37°C and washed twice with TBST (each for 15 min). Then the slides were visualized with 3,3’-diaminobenzidine (DAB) (Maixin, Fujian, China) for 5 min and counterstained with haematoxylin for 90 seconds. The slides were mounted and photographed with Olympus BX51 microscope (Olympus, Japan).

### Evaluation of staining

The slides were evaluated by two independent pathologists blinded to clinicopathologic features and clinical course under a light microscope (BX51, Olympus, Japan). Bak staining intensity was scored as 0 (negative,-), 1 (weak, +), 2 (moderate, ++), and 3 (strong, +++). The extent of staining was scored as 0~1.0 (0%~100%). The final staining score (0–3) was calculated as the multiplication of the intensity score and extent score. The final score ≥1 was defined as high expression, otherwise was defined as low expression.

### Western blot

Cells were collected and resuspended in RIPA lysis buffer for 60 min incubated on ice. After centrifuging for 20 min at 12000g and 4°C, the supernatant was collected. Protein concentrations were determined by using Bicinchoninic Acid Protein Assay kit (Thermo Fisher Scientific, Rockford, IL, USA). 60 μg of protein was separated on a 10% SDS-PAGE gel and transferred onto a polyvinylidene difluoride (PVDF) membrane (Millipore, Billerica, MA, USA). The membrane was rinsed with TBST and incubated in 5% nonfat milk diluted with TBST at 37°C for 2 h. The membrane was then incubated with mouse anti-Bak (1:1000 dilution, Cell signaling technology), mouse anti-c-PARP (1:1000 dilution, Cell signaling technology), and mouse anti-GAPDH primary antibodies (1:3000 dilution, Santa Cruz, Dallas, Texas, USA) respectively overnight at 4°C. After washing with TBST 3 times for 15 min each time, the membrane was incubated with the appropriate secondary antibodies for 1 hour at 37°C. The membranes were then washed with TBST 4 times for 10 min each time and visualized the bands by using an ECL kit (Millipore, Billerica, MA) in a Gel Doc™ XR+ and ChemiDoc™ XRS+ System (BioRad, Hercules, CA). Image-Pro plus software 6.0 (Media Cybernetics, Rockville, MD, USA) was used to analyze the relative protein expression which was normalized to GAPDH.

### MTT assay

Cell proliferation was measured by MTT assay. For MCF-7 cells with overexpression of Bak, 5000 cells were seeded into a 96-well plate and incubated with or without 400 nM Taxol (Corden Pharma Latina S.P.A, Sermoneta, Latina, Italy) for 0, 24, 48, 72 and 96 h at 37°C with 5% CO_2_ respectively; for MDA-MB-231 cells with knockdown of Bak, 5000 cells were seeded into a 96-well plate and incubated with 0, 40 or80 nM Taxol for 48h at 37°C with 5% CO_2_. At the indicated time point, 200μl MTT (Sigma-Aldrich, St. Louis, MO, USA) at a 0.5 mg/ml final concentration was added into each well and incubated for 4 h at 37°C. The MTT medium was removed and 150 μl of DMSO was added to each well. After 15 min at room temperature, the optical density (OD) at 490 nm was detected by using a PARADIGM Detection Platform (Beckman, Brea, CA). This assay was repeated 3 independent times.

### Cell apoptosis assay

For MCF-7 cells with overexpression of Bak, cells were divided into four groups: NC, NC+Taxol, Bak, and Bak+Taxol. MCF-7 cells were seeded into a 6-well plate and incubated with or without 400 nM Taxol for 48 h at 37°C with 5% CO_2_ respectively; for MDA-MB-231 cells with knockdown of Bak, cells were seeded into a 6-well plate and incubated with 80 nM Taxol for 48 h at 37°C with 5% CO_2_ respectively, and scramble siRNA served as a negative control. For the cell apoptosis assay, Annexin V-FITC/PI Apoptosis Detection Kit (Cat: KGA108, KeyGEN Biotech, Nanjing, China) was used. According to the manufacture instruction, 10^6^ cells were collected and washed with PBS twice and then resuspended in 500 μl 1 ×binding buffer. 5 μL Annexin V-FITC and 5 μL PI were added, mixed well, and incubated for 15 min protected from light. Cells were analyzed by using MoFo High Performance Cell Sorter (Beckman, Brea, CA). This assay was repeated 3 independent times.

### Cell cycle analysis

Cells stably transfected with Bak or vector were seeded into a 6-well plate and incubated with or without 400 nM Taxol for 24 h at 37°C with 5% CO_2_. Cells from each group were trypsinized and washed with cold PBS. Following fixation in 75% alcohol for 16 h at 4°C, cells were washed with cold PBS and treated with ribonuclease A (TaKaRa, Japan) for 15 min at 37°C and then stained by prodiumiodide (Cat: BA11100, BioBox, Nanjing, China) for 15 min protected from light. The cell cycle was analyzed by using MoFo High Performance Cell Sorter (Beckman, Brea, CA). The experiments were independently performed in triplicate.

### Statistical analysis

GraphPad Prism 5 software (Graphpad Software, Inc., La Jolla, CA, USA) was used to perform statistical analysis. The data are presented as the mean ± standard deviation. The contingency data was analyzed by using a chi-squared or Fisher’s exact test. The OS estimates over time were calculated using the Kaplan-Meier method with log-rank test. Student’s *t*-test and one-way ANOVA were also used depending on experimental conditions. A value of *P*<0.05 was considered to be statistically significant.

## Results

### Association between the expression of Bak proteins and clinicopathological features of breast cancer

**Fig 1:**
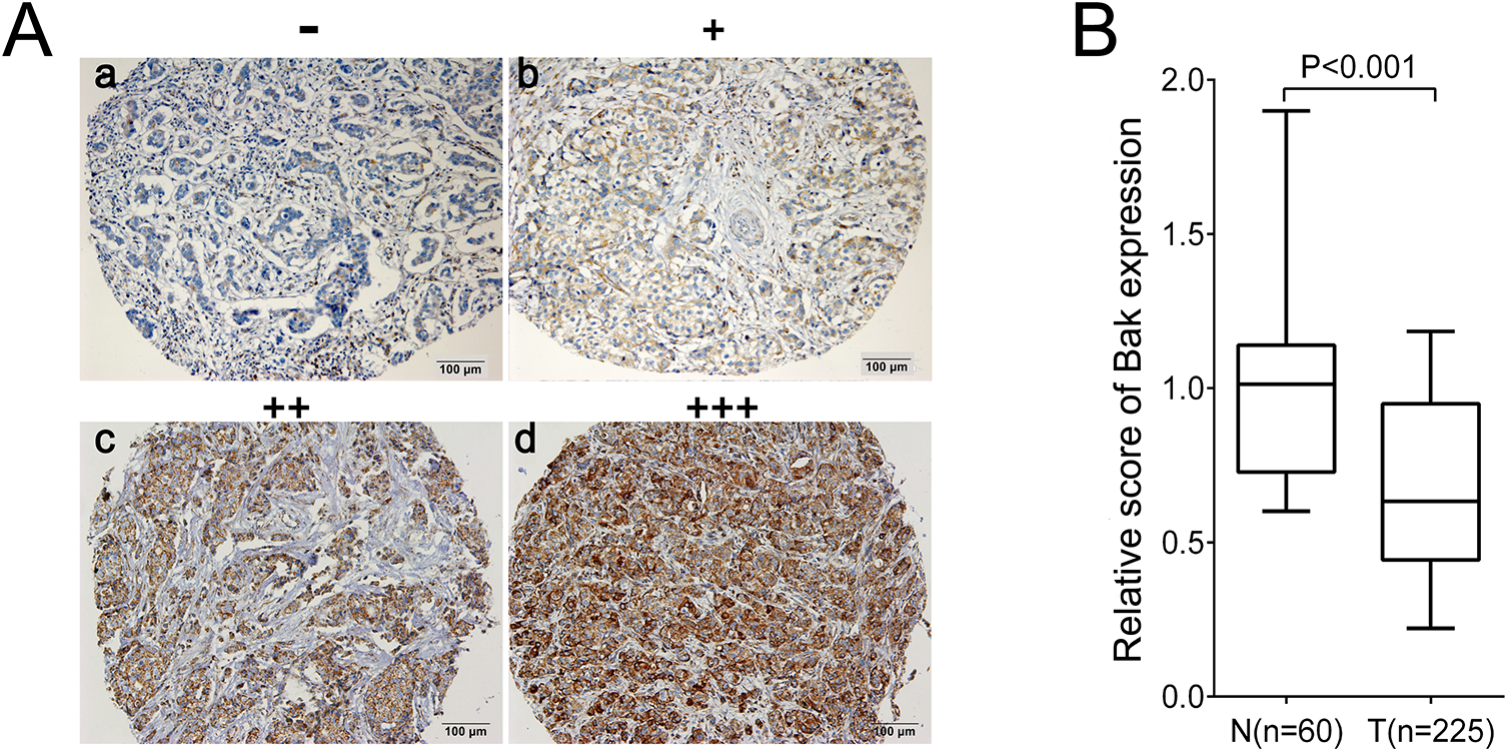
Evaluation of Bak expression in breast cancer and non-cancerous breast control tissues. (A) Representative images of Bak expression with negative (-) (a), weak (+) (b), moderate (++) (c), and strong (+++) (d) staining. Bar = 100μm. (B) Relative score of Bak expression in breast cancer and non-cancerous breast control tissue. N, non-cancerous; T, tumor tissue; n, number of case.

We detected the positive expression of Bak in breast cancer and non-cancerous breast control tissue by IHC. Expression of the Bak protein found in the cytoplasm of breast cancer cells was scored as negative (-), weak (+), moderate (++), and strong (+++) (Fig 1A). The percentage of high Bak expression in the breast cancer and the noncancerous breast control tissue was 58.7% (132/225) and 85.5% (53/62) respectively. There was a significantly lower score of the Bak protein expression in breast cancer compared with that of non-cancerous breast control tissue (P< 0.001) (Fig 1B).

**Fig 2:**
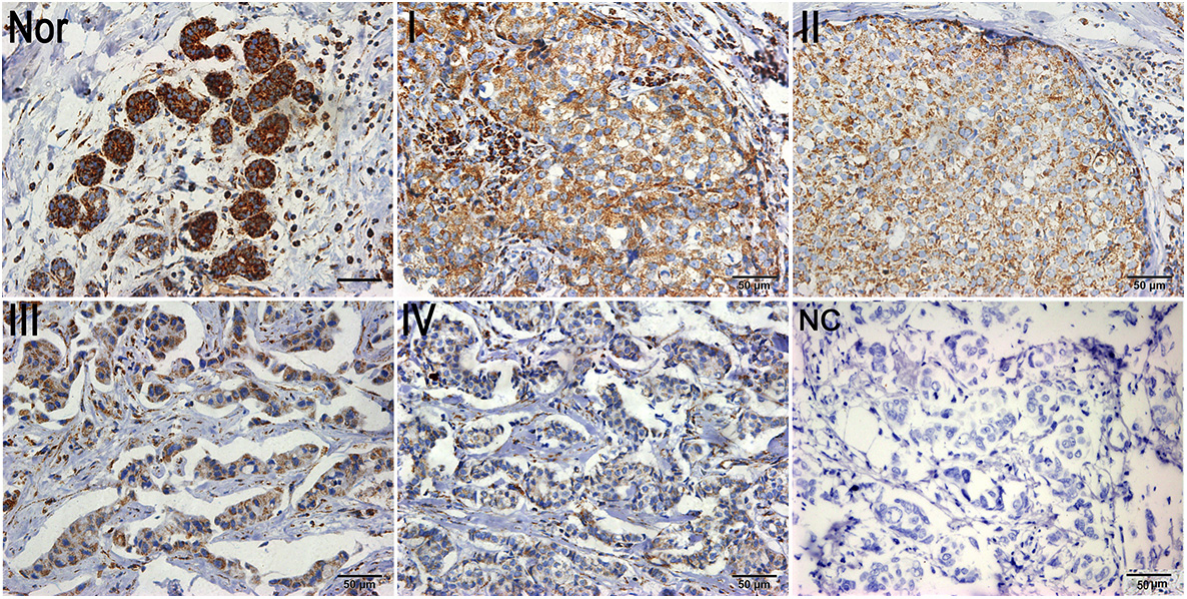
Representative images of Bak expression in different TNM stages. Representative images of Bak expression in normal breast control tissues (Nor), stage I (I), stage II (II), stage III (III), and stage IV (IV) with negative control (NC). Bar = 50 μm.

We further investigated the association between the expression of the Bak protein and clinicopathological features of breast cancer including age, gender, tumor size, lymph node metastasis status, distant metastasis, and clinical TNM stage in a univariate chi-square test. As shown in Table 2, no significant differences were observed between Bak protein expression and age, gender, lymph node metastasis status, or distant metastasis of breast cancer (P>0.05). However, Bak expression was significantly associated with the tumor size (P = 0.014). The percentage of breast cancer patients with high expression of the Bak protein was decreased as the clinical TNM stage increased whereas the percentage of those with low expression of the Bak protein exhibited the opposite correlation (P for trend = 0.005). Moreover, the IHC results also showed that the expression of Bak was reduced with an increase in the clinical TNM stage of breast cancer patients (Fig 2). No significant correlation was discovered between expression of Bak and expression of ER, PR and HER2 in breast cancer patients (P>0.05).

**Table 2:** Association between Bak expression and clinicopathologic characteristics in breast cancer.

	Bak expression		χ^2^ test
Variables	High (score ≥ 1) n = 132	Low (score<1) n = 93	P
Age	46.13 ± 0.92	46.73 ± 0.94	
Sex (%)			0.4
Male	1(100%)	0(0)	
Female	131(58.5%)	93(41.5%)	
Tumor size (%)			0.014*
T1-T2	84(66.1%)	43(33.9%)	
T3-T4	48(49.0%)	50(51.0%)	
Nodal metastasis (%)			0.058
Present	106(62.4%)	64(37.6%)	
Absent	26(47.3%)	29(52.7%)	
Distant metastasis (%)			0.073
Present	10(41.7%)	14(58.3%)	
Absent	122(60.7%)	79(39.3%)	
TNM stage (%)			0.005**
I	18(81.8%)	4(18.2%)	
II	74(60.6%)	48(39.4%)	
III	30(52.6%)	27(47.4%)	
IV	10(41.7%)	14(58.3%)	
ER expression (%)			0.83
Positive	70(50.8%)	48(54.6%)	
Negative	62(49.2%)	45(45.4%)	
PR expression (%)			0.35
Positive	64(46.0%)	51(54.5%)	
Negative	68(54.0%)	42(45.5%)	
HER2 expression (%)			0.07
Positive	84(61.9%)	48(54.6%)	
Negative	48(38.1%)	45(45.4%)	

**Fig 3:**
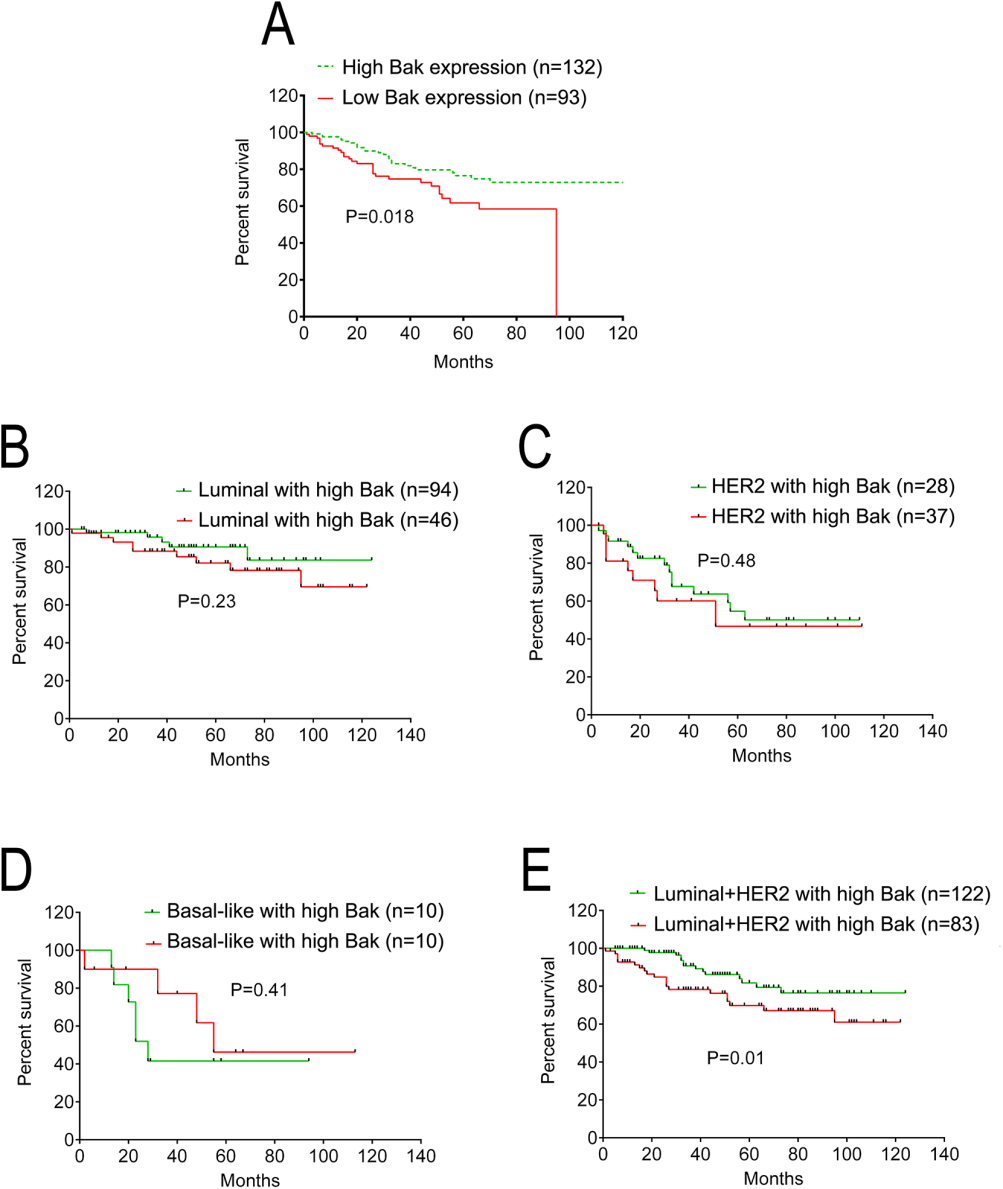
Kaplan-Meier overall survival curves of breast cancer patients and their expression of the Bak protein. Kaplan-Meier analysis plotting the survival curve of 225 cases of breast cancer with the expression of Bak and clinicopathological characteristics with statistical significance being assessed using the log-rank test. (A) Kaplan-Meier curves showed worse overall survival rates for breast cancer patients with low Bak expression compared with patients with high Bak expression (P = 0.018). (B) Patients with high Bak expression had slight but not significant higher overall survival rates than those with low Bak expression in Luminal subtype (P = 0.23). (C) Patients with high Bak expression had slight but not significant higher overall survival rates than those with low Bak expression in HER2 subtype (P = 0.48). (D) Patients with high Bak expression had a comparable overall survival rate compared with those with low Bak expression in Basal-like subtype (P = 0.41). (E) Patients with high Bak expression had significantly higher overall survival rates than those with low Bak expression in Luminal plus HER2 subtypes (P = 0.01).

We further analyzed the association between Bak expression and clinical outcome. All 225 breast cancer patients were included in the survival curves. The median OS time was 41 months with a range of 2 to 121 months. The OS rate of patients with high Bak expression was significantly increased compared with the survival of patients with low Bak expression (P = 0.018, Hazard Ratio = 1.946, 95% CI of ratio: 1.122 to 3.377, Fig 3A). To investigate the association between Bak and molecular subtypes of breast cancer, all of the 225 breast cancer cases were classified into the following groups: luminal (high Bak expression, n = 46; low Bak expression, n = 94) identified as ER+ and/or PR+, HER2 (high Bak expression, n = 28; low Bak expression, n = 37) identified as ER/PR- and HER2+, Basal-like (high Bak expression, n = 10; low Bak expression, n = 10) identified as ER/PR- and HER2- [13]. There was no significant difference between the OS rate of patients with high Bak expression and that of patients with low Bak expression in molecular subtypes of Luminal (Fig 3B), HER2 (Fig 3C), or Basal-like (Fig 3D) respectively. Interestingly, the OS rate of patients with high Bak expression was significantly increased compared with the survival of patients with low Bak expression when the Luminal and HER2 subtypes were combined (P = 0.010, Hazard Ratio = 0.488, 95% CI of ratio: 0.2461 to 0.9672, Fig 3E) indicating a close relationship between Bak and hormone/HER2 receptors.

### High Bak expression has a favorable prognosis in breast cancer patients treated with Taxol

**Fig 4:**
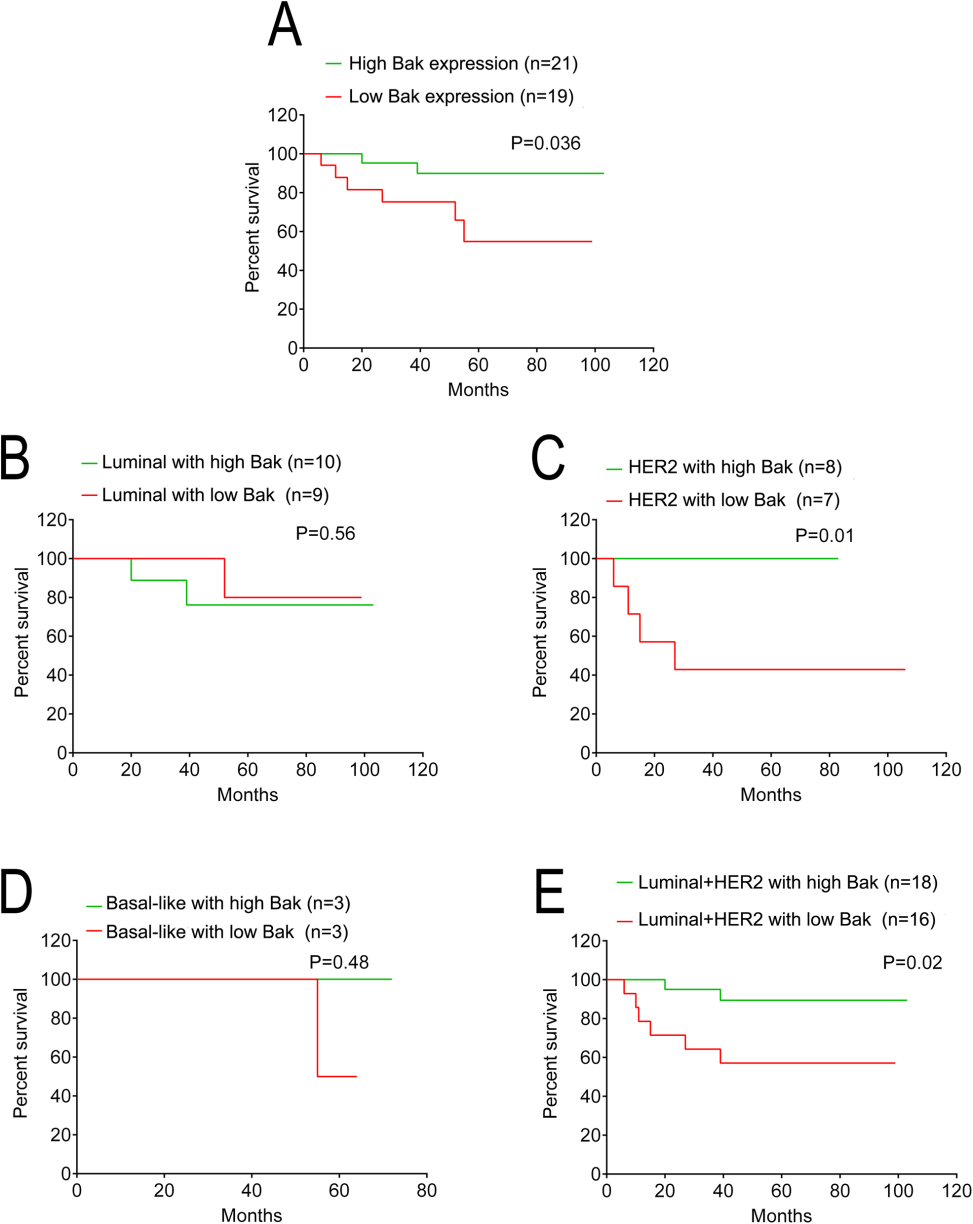
Kaplan-Meier overall survival curves of breast cancer patients treated with Taxol and their expression of the Bak protein. (A) Kaplan-Meier curves showed worse overall survival rates for breast cancer patients treated with Taxol with low Bak expression compared with patients with high Bak expression (P = 0.036). (B) Patients with high Bak expression had no significantly different overall survival rates compared with those with low Bak expression in Luminal subtype (P = 0.56). (C) Patients with high Bak expression had significantly higher overall survival rates than those with low Bak expression in HER2 subtype (P = 0.01). (D) Patients with high Bak expression had no significantly different overall survival rates compared with those with low Bak expression in Basal-like subtype (P = 0.48). (E) Patients with high Bak expression had significantly higher overall survival rates than those with low Bak expression in Luminal plus HER2 subtypes (P = 0.02).

To investigate the relationship between Bak and Taxol treatment in breast cancer, 40 cases that used therapeutic strategies containing Taxol were selected from the 225 breast cancer patients. As shown in Table 3, no significant differences were observed between expression of Bak proteins and age, gender, lymph node metastasis status, or distant metastasis of breast cancer patients treated with Taxol (P>0.05). However in line with the results above, Bak expression was significantly associated with the tumor size (P = 0.026). The percentage of patients with high expression of the Bak protein was decreased with an increase in the clinical TNM stage, and the percentage of those with low expression of the Bak protein exhibited the opposite correlation (P for trend = 0.03). Furthermore, high Bak expression indicated a higher OS rate in patients treated with Taxol compared with those of patients with low Bak expression (P = 0.036, Hazard Ratio = 5.021, 95% CI of ratio: 1.134 to 22.24, Fig 4A). Moreover, there was no significant difference between the OS rate of patients with high Bak expression and that of patients with low Bak expression in molecular subtypes of Luminal (Fig 4B) or Basal-like (Fig 4D) treated with Taxol respectively, but the OS rate of patients with high Bak expression was significantly increased compared with the survival of patients with low Bak expression in HER2 subtypes treated with Taxol (P = 0.010, Hazard Ratio = 0.07, 95% CI of ratio: 0.0095 to 0.5424, Fig 4C). Additionally, the OS rate of patients with high Bak expression was significantly increased compared with the survival of patients with low Bak expression when the Luminal and HER2 subtypes were combined (P = 0.02, Hazard Ratio = 0.18, 95% CI of ratio: 0.04 to 0.77, Fig 4E).

**Table 3:** Association between Bak expression and clinicopathologic characteristics in breast cancer treated with Taxol.

	Bak expression		χ^2^ test
Variables	High (score ≥ 1) n = 21	Low (score <1) n = 19	P
Age	44.48 ±1.97	47.97 ±1.78	
Sex (%)			
Male	0(0%)	0(0%)	
Female	21(52.5%)	19(47.5%)	
Tumor size (%)			0.026*
T1-T2	20(66.7%)	13(33.3%)	
T3-T4	1(31.2%)	6(68.8%)	
Nodal metastasis (%)			0.451
Present	17(50.0%)	17(50.0%)	
Absent	4(66.7%)	2(33.3%)	
Distant metastasis (%)			0.12
Present	1(25.0%)	4(75.0%)	
Absent	20(57.1%)	15(42.9%)	
TNM stage (%)			0.03*
I	2(100.0%)	0(100.0%)	
II	14(63.6%)	8(36.4%)	
III	2(28.6%)	5(71.4%)	
IV	3(33.3%)	6(66.7%)	

### Induced Bak expression sensitizes breast cancer cells to Taxol

**Fig 5:**
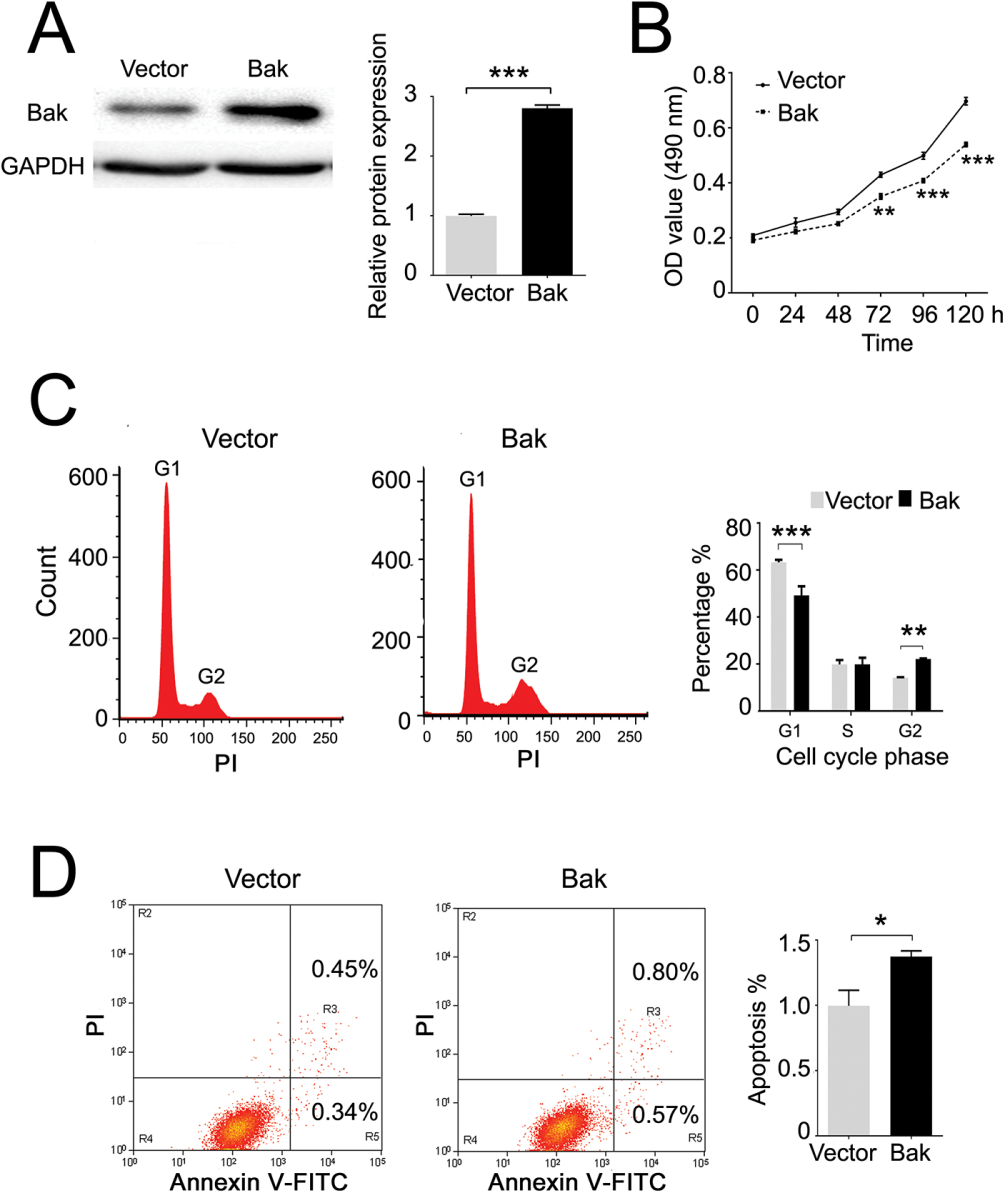
Overexpression of Bak in MCF-7 cells reduces cell growth and arrests the cell cycle at G2/M phase. (A) Western blot detection of the Bak expression after transfection and its quantification. (B) Overexpression of Bak reduced the cell growth measured by MTT assay. (C) Overexpression of Bak arrests the cell cycle at G2/M phase measured by cytometry analysis. (D) Significant changes were observed in apoptosis between the vector control and Bak groups measured by cytometry analysis. Data are expressed by means ± sd. *P<0.05, **P<0.01, ***P<0.001 *vs* vector control.

**Fig 6:**
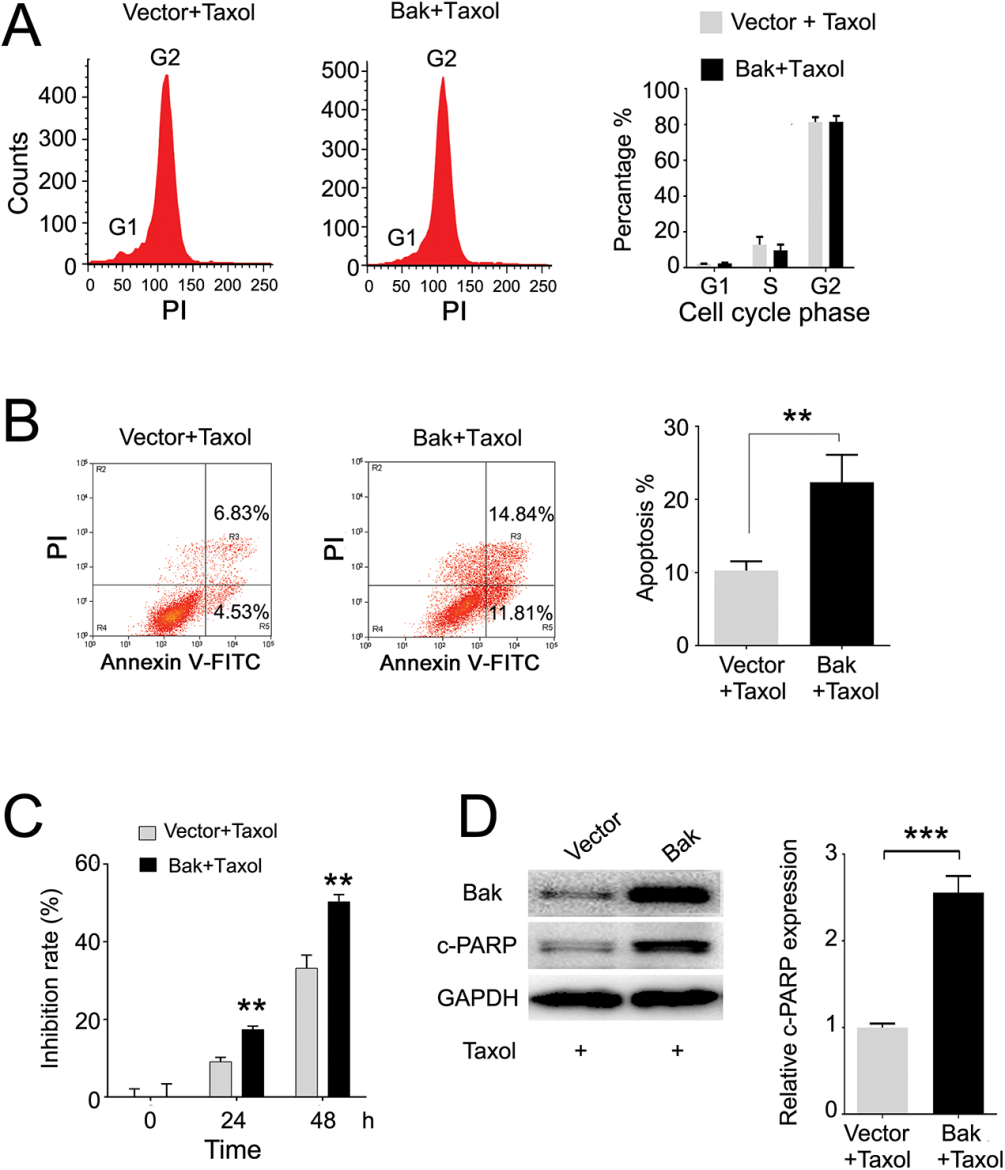
Bak sensitizes MCF-7 cells to Taxol by inducing apoptosis. (A) Taxol arrested the cell cycle at G2/M phase measured by cytometry analysis with no significant difference between the two groups. (B) Bak sensitized MCF-7 cells to Taxol by increasing apoptosis measured by cytometry analysis. (C) Bak significantly increased the inhibition rate compared with the vector control group. (D) Western blot detection of the Bak and c-PARP expression after Taxol treatment and its quantification. c-PARP, cleaved PARP. Data are expressed by means ± sd. **P<0.01, ***P<0.001 *vs* vector control.

**Fig 7:**
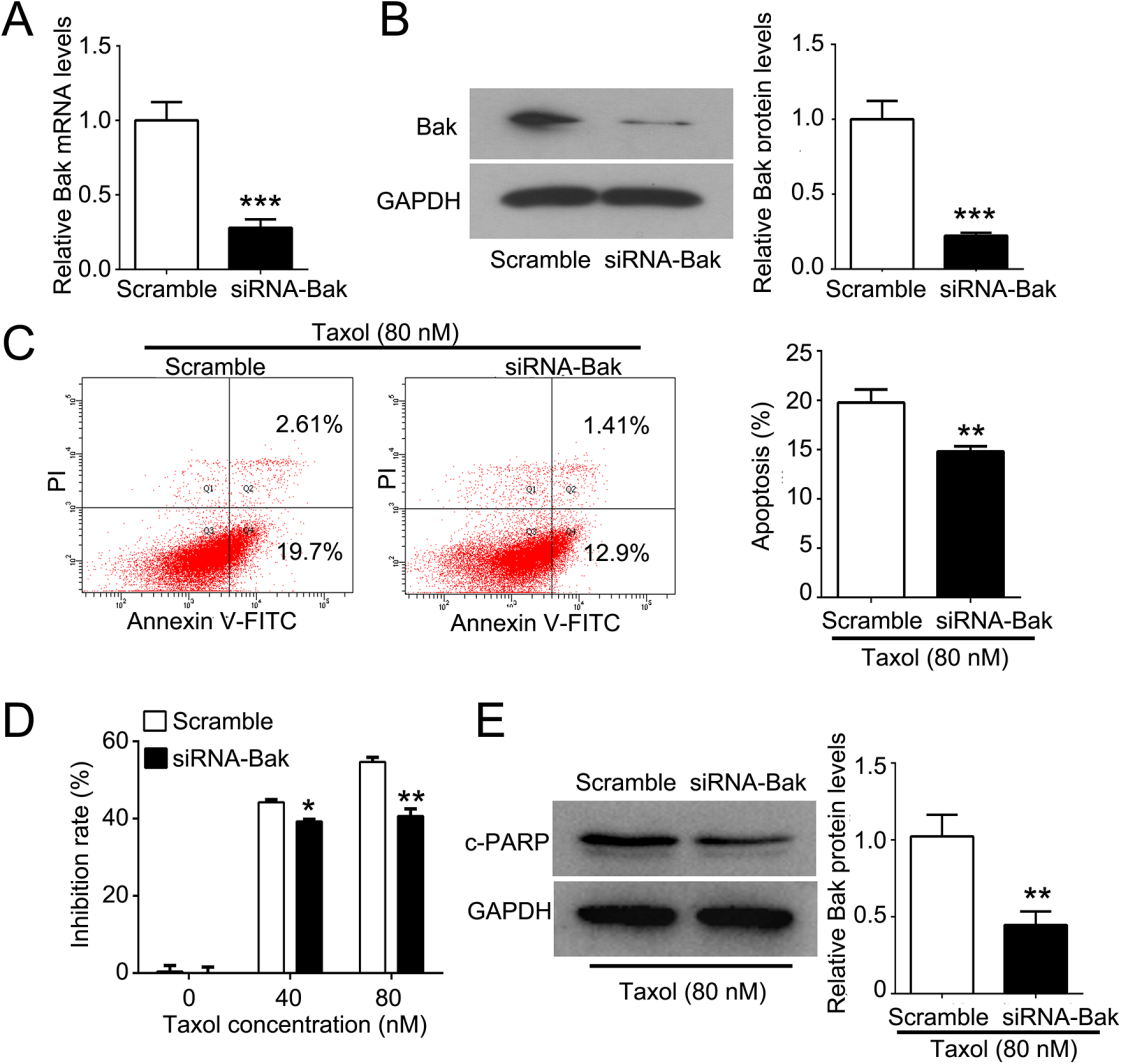
Downregulation of Bak confers resistance of MDA-MB-231 cells to Taxol. (A) QPCR detection of Bak mRNA after siRNA transfection. (B) Western blot detection of the Bak expression after siRNA transfection and its quantification. (C) Downregulation of Bak inhibited Taxol induced apoptosis in MDA-MB-231 cells measured by cytometry analysis. (D) Downregulation of Bak significantly decreased the inhibition rate compared with the negative control group. (E) Western blot detection of c-PARP expression and its quantification. c-PARP, cleaved PARP. Data are expressed by means ± sd. **P<0.01, ***P<0.001 *vs* negative control.

To test if high Bak expression can sensitize tumor cells for breast cancer patients treated with Taxol, the Bak gene was stably transfected into MCF-7 cells indicated by significant upregulation of Bak (Fig 5A). Upregulation of Bak significantly inhibited cell proliferation at 72, 96, and 120 hours compared with the control (Fig 5B). Furthermore, overexpression of Bak induced cell cycle arrest at the G2/M phase (Fig 5C) and increased the apoptosis percentage by approximate 30% (Fig 5D). Since high Bak expression had a higher OS in patients treated with Taxol compared with the survival of patients with low Bak expression, we next investigated the role of Bak in breast cancer cell undergoing a Taxol challenge. By flow cytometry analysis, we found that induced Bak expression significantly promoted MCF-7 cell apoptosis after Taxol treatment compared with the control by two-fold (Fig 6B), but there was no significant alteration on the G2/M phase cell cycle arrest (Fig 6A). We also found that the proliferation of cells treated 400 nM Taxol was dramatically inhibited in the Bak transfection group compared with the vector control group in a time dependent manner (Fig 6C). In addition, the cleaved-PARP (c-PARP, apoptosis marker) was significantly increased in Bak overexpressed MCF-7 cells compared with that of the vector control after treatment with Taxol for 48 h (Fig 6D). Furthermore, we knocked down the mRNA and protein levels of Bak in MDA-MB-231 cells (Fig 7A and 7B). We found that downregulation of Bak inhibited Taxol induced apoptosis and cell cytotoxicity (Fig 7C and 7D). The c-PARP was significantly decreased in MDA-MB-231 cells with downregulation of Bak compared with negative control after treatment with 80 nM Taxol for 48 h (Fig 7E).

## Discussion

Although improvements have been made at the early stage of chemotherapy in breast cancer, a large proportion of patients are non-responsive to current treatment strategies due to drug resistance, such as Paclitaxel [14], highlighting the necessity to develop strategies to overcome or bypass drug resistance. Because the suppressive role of Bak has been commonly recognized in molecular, cellular, and animal models [15], it is imperative to illustrate the association between Bak expression and clinical features and therapy strategies. In our recent study, a total of 225 cases of breast cancer tissues with detailed clinical characteristics were collected from 2001 to 2012 and constructed into tissues microarrays. The expression of Bak was evaluated by IHC. We found that the percentage of cases with high Bak expression was significantly increased in early stage breast cancer patients (stage I~II) with higher overall survival and longer survival time compared with that of late stage breast cancer patients (stage III~IV). Furthermore, the high Bak expression in the patients treated with Taxol exhibited a favorable prognosis clinically compared with those with low Bak expression. The mitochondrial apoptotic pathway is one of the important mechanisms involved in Taxol induced apoptosis and drug resistance [16]. Accumulative evidence has demonstrated that Bcl-2 family members are important regulators of apoptosis related to chemotherapy response, outcome, and overall prognosis in breast cancer. For example, lower BCL2 expression is related with poorer clinical outcome in patients with metastatic breast carcinoma in luminal breast cancers [17] and overexpression of the BCL2 gene is found to be associated with resistance against chemotherapeutics treatment [18]. In addition, the higher protein expression of Bax is observed in lower the degree of tumor differentiation and in patients positive for lymph node metastasis and for distant metastasis, and predicts a poorer outcome [19]. The pro-apoptotic protein Bak represents the most common dysregulation in the cancer genome including breast cancer [20]. Increasing evidence suggests that the upregulation of Bak is beneficial for promoting breast cancer cell death after Taxol treatment [5].

In addition, in HER2 subtypes treated with Taxol and in Luminal plus HER2 subtypes with any therapy strategies, our recent results showed that the OS rate of patients with high Bak expression was significantly increased compared with the survival of patients with low Bak expression. Neoadjuvant chemotherapy is considered to be capable of improving survival particularly in patients with HER2+ breast cancers which may increase pathologic complete response rates. It was shown that 44% of HER2+ tumors and 31% of HER2+ plus hormone receptor+ tumors responded with pathologic complete response in patients who received anthracycline-free neoadjuvant chemotherapy with carboplatin and Paclitaxel with or without trastuzumab [21]. A randomized phase II study suggests a numerically modest pathological complete response rate increase with a double anti-HER2 blockade plus chemotherapy containing Paclitaxel [22]. Miura D et al. demonstrated that adding Paclitaxel to trastuzumab significantly enhanced antibody-dependent cell-mediated cytotoxicity with levels twice as great as with trastuzumab monotherapy through a rapid recruitment of NK cells [23]. A very recent study shows that treatment with adjuvant Paclitaxel plus trastuzumab is associated with a risk of early recurrence of only about 2% among women with predominantly stage I HER2-positive breast cancer [24]. The effects of the HER2 antibody on cell death are reversed by chemicals that regulate the pro-apoptotic channels in the mitochondria and lacking Bak generates resistance to the deleterious effects of the HER2 antibody [25]. Bean GR et al. demonstrated that PUMA and BIM were the key apoptotic effectors of tyrosine kinase inhibitors in breast cancers with overexpression of HER2 by directly activating Bak to permeabilize mitochondria leading to caspase activation and apoptosis [26]. Previous studies show that reduction of Bak was involved in the malignant development of breast cancer in both hormone-dependent and hormone-independent manners [27–29]. These findings suggest that Paclitaxel is effective in HER2-positive and hormone receptor-positive breast cancer which may be activating Bak to permeabilize mitochondria.

Mitochondrial pathway is one of important death signals. This pathway is initiated by stress, mitotic inhibitors, or exposure to cytotoxic agents such as Paclitaxel. These stimulations trigger mitochondrial outer membrane permeabilization, which is dependent on the activation of BCL2 effector protein, BAK. The activated-Bak releases from protective BCL-2 proteins, and then perturbs the mitochondrial membrane forming pores that permit release of cytochrome c, leading to the activation of caspase 9, and ultimately apoptosis [30]. Deficiency of Bak expression is frequently observed in gastric cancer [31], colorectal cancer [32], and prostate cancer [33] with a close correlation between occurrence and development of tumors. Previous studies showed that Bak activation could inhibit cell growth in hepatocellular carcinoma, prostate cancer and glioma [34–36], and Bak deletion could stimulate gastric epithelial proliferation [37].We here found that upregulation of Bak in MCF-7 cells dramatically arrested the cell cycle during G2/M phase and sensitized these cells to Taxol by inhibiting proliferation and promoting apoptosis *in vitro*. In addition, we found that Bak overexpression arrested breast cancer cells at G2/M phase which did not induce a strong cellular apoptosis but might accumulate an apoptotic signal. Upon stimulated with the apoptotic signal as Paclitaxel, Bak overexpression induced a significantly higher apoptosis than that of control cells. By using loss of function experiments, Anna V. Miller et al. illustrated that Paclitaxel-induced apoptosis was Bak-dependent and associated with interaction between Bak and MCL-1 in breast tumors [10]. Our previous study showed that Bak as a direct target of miR-125b was downregulated in Taxol-resistant breast cancer cells, and overexpression of Bak by knockdown of miR-125b could overcome Taxol resistance [11]. In this study, we also showed that downregulation of Bak through siRNA transfection inhibited Paclitaxel induced apoptosis in breast cancer cells. Taken together, these results suggest that Bak inhibits cell proliferation by arresting cell cycle, and sensitizes breast cancer cells to Paclitaxel by enhancing apoptosis.

In conclusion, we report here that high Bak expression is associated with the favorable overall survival rates of breast cancer and also correlated with the sensitivity of Taxol chemotherapy in breast cancer patients. Direct upregulation of Bak or indirect alteration of its related interaction molecules will be therapeutically beneficial for overcoming Taxol resistance in breast cancer particularly in HER2-positive and hormone-positive cases.
